# Maternal perception of breastfeeding in children with unilateral cleft lip and palate: A qualitative interpretative analysis

**DOI:** 10.1186/s13006-022-00528-y

**Published:** 2022-12-19

**Authors:** Cerón-Zapata Ana María, Martínez-Delgado Cecilia María, Calderón-Higuita Gloria Emilia

**Affiliations:** 1 Universidad CES, Dental School, Calle 10 A #, 22-04 Medellín, South America Colombia; 2 International Board Certified Lactation Consultant® (IBCLC® L-70587), Medellín, Colombia

**Keywords:** Perception, Experiences, Breastfeeding, Cleft lip and palate, Presurgical orthopedics, Nasoalveolar molding

## Abstract

**Background:**

Unilateral cleft lip and / or palate (UCL/P) is one of the most common congenital craniofacial differences. The objective of this study was to describe the maternal perception of breastfeeding in children with unilateral cleft lip and palate and to assess the role of breastfeeding counseling.

**Methods:**

This study was conducted using an interpretive phenomenological approach to explore the experience from the perspectives of mothers breastfeeding her UCL/P child. Twenty-eight mothers of patients with nonsyndromic UCL/P treated with nasoalveolar molding (NAM) therapy between April 2015 and April 2018 were selected during consultations at the Fundación Clínica Noel in Medellín, Colombia. Thematic analysis was conducted for qualitative data.

**Results:**

The findings resulted in six main categories: First contact with the CL/P team, access to early diagnosis and timely treatment, perceptions of parents about health personnel on breastfeeding of CL/P patients, perceptions of mothers toward breastfeeding, perception of advantages and disadvantages of the NAM technique regarding breastfeeding and assessment of the CL/P team. The interviewed mothers, both prenatally and postnatally, stated the benefits of initiating the process prenatally. There are still difficulties in reaching a timely diagnosis. Several mothers noted that health professionals and assistants determined the hospitalization, installation of a nasogastric tube or feeding through a baby bottle or syringe, which prevented the first contact between mother and child. Even though the exclusive breastfeeding process is difficult for these mothers, they acknowledge its immense advantages. Interviewed mothers considered using the NAM therapy advantageous as the obturator allowed a better bottle-feeding process. The interdisciplinary team generates satisfaction, motivation, expectations and happiness in the mothers who initiated this therapy with their children.

**Conclusion:**

The participants related difficulties with exclusive breastfeeding. Mothers acknowledged the clinical results when using the NAM therapy and the support provided by the IBCLC. We encourage health providers in Medellín, Colombia, to seek education to enhance their clinical skills and promote and protect breastfeeding. Also, health professionals in other cities or countries could consider search more breastfeeding education as well.

## Background

Cleft lip and/or palate (CL/P) is one of the most common congenital craniofacial differences and may produce these patients’ aesthetic, functional and emotional disorders [1]. It shows a prevalence of one per 1000 live births [2]. The prevalence in Colombia is 2,78 per 10.000 inhabitants [3]. This condition affects the patient and his/her family causing difficulties in emotional and social development [4], feeding [5], breathing and the development of speech [6], occlusion [7] and physical appearance [8]. Therefore, during the parents’ first acknowledgment with the team is important to understand and assume that having a child with CL/P can be difficult to cope [4].

Exclusive maternal breastfeeding [9] is the best option to ensure that the infant receives the essential nutrients needed for growth and development during the first 6 months of life [10]. In addition, breast milk is the main choice to prevent adverse health conditions and promote intellectual and language development [11]. One of the complications exhibited by children with CL/P is feeding. An inability to create a seal around the nipple during breastfeeding has been observed, leading to failure to generate enough negative intraoral pressure during suction [12]. This situation could lead to inadequate nutrient intake and cause the newborn’s growth and development delay [13].

Current advances in pre-surgical orthopedic treatment protocols for CL/P, including nasoalveolar molding (NAM) obturators, are an alternative for improving the quality of the reparation process since they actively improve the affected nostril and passively the alveolar segments [14–16]. However, the treatment protocol has observed that breastfeeding still presents difficulties, some arising from mother’s perception, such as discomfort to the infant and the idea that the child is still hungry [17]. Even though research, including quantitative approaches, has focused on the first reactions to the diagnosis and measurement of stress levels during the first years of life [18, 19], qualitative investigations that assess mothers’ perception of breastfeeding and CL/P are scarce.

Therefore, this investigation aims to describe maternal perception of exclusive breastfeeding in children with unilateral cleft lip and palate (UCL/P) and to assess the role of breastfeeding counseling with an International Board of Certified Lactation Consultant (IBCLC).

## Methods

This study was conducted to answer the research question: ¿What is the maternal perception experienced of mothers exclusive breastfeeding unilateral cleft lip and palate infant treated with NAM therapy, after breastfeeding counseling with an IBCLC? An interpretive phenomenological approach was used to explore the experience from the perspectives of mothers breastfeeding their CL/P child [20–22].

All mothers were born in Colombia, as different cultures have different attitudes towards CL/P and the authors wished to focus on issues relating to Colombian culture. This research is based on a group of Colombian heterosexual women and thus, interpretations presented cannot be automatically generalized to the population.

### Eligibility criteria

Potential participants were recruited when they arrived to consult with the cleft palate team. The researchers gave the study information to the mothers. One of the researchers was the treating pediatric dentist.

The sample included mothers of patients between 0 and 2 months of age with either prenatal or postnatal diagnosis of non-syndromic UCL/P. Mothers were excluded from selection if they could not attend scheduled periodic appointments, if they did not speak Spanish, if their infant had a congenital abnormality likely to affect feeding or if their infant was considered gravely ill.

Before the interviews, mothers received orientation by an International Board Certified Lactation Consultant® (IBCLC International®). She did a breastfeeding personal education program that provided expert breastfeeding and lactation care to CL/P patients’ needs, promoted changes that support breastfeeding and helped mothers with their questions. Human milk (HM) expression was encouraged in mothers who could not breastfeed. The pediatric dentist explained the NAM treatment and detailed oral hygiene and diet counseling. Their UCL/P’s children received treatment with NAM.

Mothers were divided into two groups because they came to the CLP team before or after the baby was born, depending on their early diagnosis and referral to the cleft members. The first group received prenatal information about the process of maternal breastfeeding from an IBCLC using a personal education program. The postnatal group received information in the same way by an IBCLC using an individual education program since they could not arrive at the CL/P team prenatally.

### Data collection

Data was generated through unstructured individual interviews. The interviews were done in private interview rooms at the clinic. Two field worker training sessions covered research ethics, obtained informed consent and data collection, with the strongest focus being on developing qualitative interview skills. The researchers applied no interview schedule. They used a map of keywords to bring up new ideas and areas for further discussion.

Fieldworkers underwent extensive practice conducting interviews after babies were born and their mothers received NAM treatment, detailed oral hygiene and breastfeeding counseling using role-plays and applying the pilot test. Four pilot interviews were carried out before the interviewer could strive to vary the focus of the phenomena under study. During data collection, researchers listened to interviews on an ongoing basis to monitor the quality of interview skills and feedback given to fieldworkers as necessary. Mothers were encouraged to share their stories in their own way, proceeding on their terms while describing aspects important to their own experience [22].

The interviews’ duration was between 60 to 90 minutes to enable spontaneity of discussion and they were audio-tape recorded, then digitized and transcribed.

Baseline data was collected using a structured quantitative questionnaire, including information about participants’ sociodemographic characteristics and infant feeding plans.

All the mothers signed the applicable informed consent before the data collection.

### Data analysis

The research group considers exclusive breastfeeding, as World Health Organization (WHO) definition, “when infant receives breastmilk expressed or from a wet nurse, included any medicine that the infant needs” [9] Mothers were asked through in-depth interview to state ¿What is her maternal perception experienced about exclusive breastfeeding unilateral cleft lip and palate infant treated with NAM, after breastfeeding counseling with an IBCLC? . An interpretive phenomenological approach was used to explore the experience from the perspectives of mothers breastfeeding their CL/P child, whether they planned to feed their baby breastmilk only, formula milk only, or both breast and formula milk. Data analysis was done using thematic analysis, which got to highlight and explores the narrated experiences, perceptions, salient events, discursive patterns and changes over time articulated by the participants [23].

To preserve anonymity, the researcher assigned codes to each participant. Audio recordings and transcripts were stored at University CES in a password-protected file.

Results were compared and minor variations were discussed, reviewed and resolved. Analysis of the interview data was based on predetermined keywords, as well as inductive themes that emerged from the analysis of the interview data [23].

The thematic analysis comprised five stages, beginning with familiarizing the transcripts to gain an overview of the content. This was followed by the development of an analytical framework based on identified research questions, as well as on themes that emerged. This framework was then applied to the individual transcripts and data charted into categories based on these identified themes. Finally, the researchers undertook a process of mapping and data interpretation [23].

The Ethics Committee of CES University approved the study through minute number 7429.

## Results

Unstructured interviews were applied to 28 mothers of children with non-syndromic unilateral cleft lip and/or palate (UCL/P), with prenatal (32%) and postnatal diagnosis (68%) mothers who consulted to receive early orthopedic treatment with NAM in the Clínica Noel Foundation interdisciplinary CL/P team program in Medellín, Colombia, between April 2015 and April 2018.

Data presented in Table 1 indicates participants’ sociodemographic characteristics.

**Table 1.** Sociodemographic characteristics of children with unilateral cleft lip and palate who participated in the study (*n* = 28)

Variable	n (%)	Median (Interquartile range)
Sex of the child		
Male	20(72%)	
Female	8 (28%)	
Age of the child (months)		
Newborn	10(36%)	
One month	6 (21%)	
Two months	8 (29%)	
Three months	4 (14%)	
Place of residence (*n* = 22)		
Urban	18(65%)	
Rural	4 (14%)	
Parents’ age (years)		
Mother		32 (21–44)
Father		33 (20–51)
Mother education (*n* = 25)		
None	7 (25%)	
Completed primary school	1 (3%)	
Completed secondary school	1 (3%)	
Technology	9 (32%)	
Completed university	7(25%)	
Father education (*n* = 24)		
None	7 (25%)	
Completed primary school	3 (11%)	
Completed secondary school	10 (36%)	
Technology	3 (11%)	
Completed university	1 (3%)	
Father occupation (*n* = 27)		
Unemployed	11 (39%)	
Self employed	1 (4%)	
Employed	5 (18%)	
Others	10 (35%)	
Place occupied by the child in the family (birth number) (n = 25)		
First	10 (36%)	
Second	5 (18%)	
Third	10 (36%)	
Skin to skin contact the first hour (*n* = 18)		
Yes	13 (46%)	
No	5 (18%)	
With the previous question, if the answer was yes, the mother can breastfeeding the baby during the first hour of life.		
Yes	5 (38%)	
No	8 (62%)	

Following/ the interpretive phenomenological approach methodology, transcripts were coded according to preliminary categories. Emerging categories were analyzed in order to compare results and reach conclusions. Table 2. The findings resulted in six main categories: 1) First contact with the CL/P team program; 2) Access to early diagnosis and timely treatment; 3) Perceptions of health personnel on breastfeeding of CL/P children; 4) Perceptions of mothers toward breastfeeding; 5) Perception of advantages and disadvantages of the nasoalveolar molding (NAM) technique regarding breastfeeding and 6) Assessment of the CL/P team program.

1. First contact with the CL/P team

**Table 2:** Summary of the categorization of the information provided by the mothers interviewed using thematic analysis

Theme	Codification	Analysis categories
Remission from the specialist	Arrival to the program	First contact with the program
Recommendation of friends		
Diagnosis pre or postnatal	Moment of diagnosis of the baby	Access to early diagnosis and timely treatment
Instructions on feeding by the health personnel at the time of the child’s CL/P birth	Contact with health personnel regarding food	Perception of health personnel to breastfeeding of CL/P patient
Feelings, difficulties, facilities of the breastfeeding process	Experiences with breastfeeding process	Perceptions about experience of breastfeeding
Ease, difficulty, advantages and disadvantages of the NAM therapy	Using the obturator with NAM therapy	Perception of advantages and disadvantages of the NAM therapy
Feeling of the process in general terms	Program score	Program evaluation

The specialist detected the anomaly during the second-trimester ultrasound referred prenatally diagnosed patients and their parents to the CL/P team program. The patients who received the diagnosis after birth were referred by friends, family members, or the medical staff that worked in the hospital where the patient was born.

The interviewed mothers, stated the advantages of initiating the process during pregnancy: minimizing the stress associated with the diagnosis from the counseling of the interdisciplinary team, psychological support, information about the procedures that the child would endure and better acceptance of the infant’s condition by the parents:

“I think it was very important because I now assimilate things better, especially the risks and benefits of the whole treatment.” [E16].

“We prepared ourselves psychologically so the impact would not be too high, right? That helped us understand the feeding procedure and the process with the obturator.” [E27].

2. Access to early diagnosis and timely treatment

There are still difficulties in reaching a timely diagnosis, especially for those people who live in rural areas or for pregnant women who access the hospital only at the time of delivery. These factors make early diagnosis difficult:

“I was referred but I had already had the baby.” [E14].

“I was referred here but the baby had already been born, he was around 3 months old.” [E20].

3. Perceptions of health personnel on breastfeeding of CL/P children

Several mothers stated that health professionals and assistants determined the hospitalization, installation of a nasogastric tube or feeding through a baby bottle or syringe, which prevented the first contact between mother and child:

“The problem with the baby was that he was in the hospital and the process stopped, it was suspended and the baby was fed through a tube.” [E2].

“To be honest, I did not breastfeed her because the cleft was wide open when she was born. She was taken to the ICU, received the tube and was left in the hospital for 10 days. I had missed the opportunity to breastfeed her, I had no milk left.” [E17].

The interviewed mothers stated that it seemed as if health professionals considered breastfeeding impossible for these patients, which made the promotion of breastfeeding difficult, despite the recommendations to breastfeed exclusively up to 6 months of age [24]:

“Well, a nurse told me that the baby had to be fed with a syringe and a baby bottle so I told her that I wanted to feed him myself and she told me that I was incapable of doing so.” [E11].

4. Perceptions of mothers toward breastfeeding

Mothers face challenges when starting the exclusive breastfeeding because children cannot effectively suck.

Bottle-feeding from an early age using breast milk from the mother can be used [12]. Mothers revealed their efforts to perform exclusive breastfeeding, their frustrations, sadness and despair caused them to be unable to breastfeed their children:

“Hard, it was very, very hard.” [E16].

“Well, sometimes you feel frustrated because you really want to breastfeed her exclusively, both for her sake and for economic reasons.” [E27].

Mothers who could start exclusive breastfeeding performed it only for a short period time. Complications arose quickly for different reasons, leading them to decide to abandon breastfeeding altogether. Mothers had the phone number’ IBCLC and they could call if they needed any support. A few called asking about manual expression and they received counseling:

“I did not have enough breast milk to feed him, although a little came out.” [E11].

“There was a time when he was malnourished or so they told me.” [E11].

“I feel like nothing comes out. I take a look at his mouth and it is dry, like he does not have enough strength to suction milk.” [E1].

Mothers stated other problems, such as inability to suction, how babies hold the nipple in their mouth, pressure from family members, fear of breastfeeding and even comparisons with previous healthy children who were able to be breastfed for periods of up to 6 months. The IBCLC gave personal education when parents need it. They could call when they need help. They received timely information and they make informed decision making:

“My baby boy could not hold my breast and I did not produce enough breast milk to feed him.” [E13].

“I was able to breastfeed my two other children, 1 up to 2 years of life and the other up to four.” [E16].

“He loved breast milk, but I had to express it because he was in the hospital for 8 days and they always fed him through a baby bottle, so I extracted it and gave it to him using the bottle.” [E23].

Hospitalization of mothers is also difficult for the exclusive breastfeeding practice as the mother-baby bond is broken. Some health services in Medellín separate the mother from the baby when she is hospitalized. This practice, sometimes led to the cessation of breastfeeding even though they received breastfeeding counseling.

From the economic standpoint, access to supplementary milk is an additional cost to mothers of low income and consumption increases as the child grows, which raises such expenses:

“We always prepared the baby bottle with the milk they recommended, but then we could not afford it anymore.” [E16].

Despite such difficulties, mothers expressed and recalled the exclusive breastfeeding as a special connection with their children and communicated their sadness because they wanted the process to last longer and be less complicated.

Even though the exclusive breastfeeding is difficult for these mothers, they acknowledge its immense advantages:

“Babies develop better with breast milk, their mental development, their hearing and vision, everything.” [E25].

“Everything, I do not know … their growth, the defense system, they are healthier.” [E25].

“Everything, every vitamin, everything.” [E26].

5. Perception of advantages and disadvantages of the nasoalveolar molding (NAM) technique regarding breastfeeding

Interviewed mothers considered using the NAM treatment disadvantageous as the obturator did not allow for the exclusive breastfeeding. The palatal cleft was narrower, a nasal molding occurred and the child gained weight:

“Now, my baby uses the baby bottle more. It is less difficult and he does not congest as much.” [E8].

“My boy has the wing of the nose uplifted, the palatal cleft is almost non-existent, which helps him a lot.” [E5].

“From the moment he received the obturator, he has gained weight and is chubbier.” [E11].

The use of the obturator for 24 hours showed no complications for any mothers. They did not perceive mood changes or sleep disorders. The cleaning process and its use, in general, were easy.

A few mothers, who continued with the exclusive breastfeeding process while using the obturator, manifested discomfort and lacerations as the main disadvantage of the obturator. Dermatitis on the child’s face was another disadvantage, but adhesives are necessary to keep the soft tissues in place and improve the position of the columella:

“When I tried to feed him using the obturator, the little wire poked me, so I decided to remove the device.” [E11].

“I was breastfeeding him before using the obturator; once he started using it, I could not feed him anymore. I had to just use the baby bottle.” [E12].

“He developed an allergy and it was horrible for him.” [E21].

The correct use of the NAM obturator therapy at home was always concerning as it was a new procedure for the mothers and they wanted to perform it correctly for the well-being of their children. One of the interviewed mothers stated:

“As a suggestion, when they give you the obturator, they should include not only general information, but also recommendations on the product for us to be able to handle the obturator correctly at home.” [E15].

6. Assessment of the CLP team program

The program generates satisfaction, motivation, expectations and happiness for the mothers who initiated this therapy with their children. All of them reported that their children made great progress:

“Wonderful! It has been a beautiful experience because when I realized that my baby was coming with this condition, I never thought that there could be a solution.” [E10].

“I am super happy. It is a great happiness because my baby has improved a lot with the obturator.” [E28].

Breastfeeding counseling provided by the researchers was valued as effective but difficult to apply daily. The mothers received additional counseling from the IBCLC when they encounter complications:

“It was effective but it was really a fallacy because there is nothing you can do when the baby is still in the womb. You have to wait until you have the baby, and what you expect is quite different from the real situation.” [E15].

Positive comments were also generated from close family members as they witnessed the progress and acceptance of the child’s condition:

“Well, they say everything is ok for the baby.” [E6].

“Everybody encourages me a lot. [E20].

### Discussion

A few qualitative studies permitted establishing similarities and differences with the results of the present investigation. Available qualitative data allows further inquiry into the experiences of individuals in specific situations, such as the condition in the current study.

This study revealed difficulties regarding exclusivity in maternal breastfeeding in a sample of UCL/P Colombian children who were treated at Clínica Noel Foundation in Medellín. Mothers positively valued the information provided by the IBCLC. However, a daily application was difficult for their. These results are in agreement with those of Lindberg and Berglund and Madhoun [17, 25] .

Some factors that discouraged maternal breastfeeding in children younger than 6 months of age included inadequate breastfeeding techniques, frequent bottle use and early introduction of complementary foods. These factors make it difficult for mothers to produce an adequate milk volume [5].

The mothers showed efforts to exclusive breastfeed their children, a product of what they learned during lactation counseling. However, they privilege the use of the feeding bottle and other techniques, like using syringes or drops, over exclusive maternal breastfeeding. This was due to suction problems, a sensation of low levels of milk in their breasts, milk coming out of the baby’s nose, social and family pressure, fear that the baby would lose weight and early hospitalization, which did not ease the mother to child bonding. Several of the reasons mentioned coinciding with a quantitative study carried out in Porto Alegre, Brazil [26] and the qualitative study by Lindberg and Berglund [25].

Garcez and Giugliani found that despite the diverse difficulties reported and the lack of professional support after discharge from the maternity wards, the initiation rate and the duration of exclusive breastfeeding of children with cleft lip and palate are compatible with successful breastfeeding [26].

Lindberg and Berglund [25] reported that, despite the difficulties, mothers were aware of the importance of breast milk, which is in agreement with the results of the current investigation, such as these Colombian mothers. Owens described the failure of mothers when trying to exclusively breastfeed their babies. Mothers found exclusive breastfeeding challenging, so they needed support, especially when babies showed additional feeding deficiencies [27]. A similar finding was reported in our study, where mothers encountered difficulties during the exclusive breastfeeding.

Amstalden Mendes et al. analyzed counseling to parents during the postnatal period, identified resources used to feed their babies and concluded a lack of attention to UCL/P patients by health professionals [28]. Amstalden Mendes et al. showed that health system factors and maternal-baby factors were the main precipitating reasons why mothers failed to breastfeed.

Regarding the NAM, Goyal et al. [29] reported between 44 and 100% breastfeeding frequency. Mothers who received counseling were more willing to breastfeed compared to mothers who did not receive it. Goyal showed that treatment with the NAM reduced breastfeeding practice. In addition, some complications were evident when using the obturator, such as the difficulty attaching the baby to the breast.

Difficult situations with the health personnel due to a negative attitude toward breastfeeding of UCL/P children at birth were reported by Lindberg and Berglund [25]. The Colombian mothers felt low medical support and a lack of breastfeeding information.

Breastfeeding siblings without UCL/P was easier in the Scandinavian study [13]. In this study, the mothers valued breastfeeding counseling, thought their siblings were easier to feed than the child with CL/P.

### Limitations

Results of qualitative research provide helpful information to generate hypotheses for future investigations, for example, related to accessibility to early prenatal diagnosis and access barriers to the health system. The experiences of these mothers might be specific to Medellín since care practices could vary widely between countries and even cities of the same country.

### Conclusions

In this study, exclusive breastfeeding children with CL/P was a difficult process, though it was vital. Thus, efforts should be directed toward a breastfeeding program implementation with pregnancy CL/P mothers, so they will have tools that allow them to deal with the expected difficulties during early breastfeeding.

In addition, it revealed that health professionals must not become an obstacle to the mother-child bonding. They should act based on attention protocols formulated according to context and culture. They must provide knowledge and support for mothers to make optimal feeding choices, increase mothers’ self-efficacy and facilitate breastfeeding success. We encourage health providers in Medellín, Colombia, to seek education to enhance their clinical skills and promote and protect breastfeeding. Also, health professionals in other cities or countries could consider search more breastfeeding education as well.

Professionals trained in breastfeeding would be a valuable resource for mothers of children with CL/P, as they support and resolve breastfeeding concerns and complications.

Another practical implication would be the encouragement to mothers who have undergone this intervention to support either in person or through video, group, or other modality new CL/P mothers prenatal and postnatal. Forming a peer-to-peer support group may help mothers cope with the complications of feeding their infants.

The design of educational strategies help mothers cope with feeding difficulties, using personal counseling, sending videos by email or WhatsApp or calling by phone. An interdisciplinary CL/P team is mandatory, especially during pregnancy, since mothers can feel better prepared to receive their children. Finally, the researchers suggest further investigation on improving accessibility to the program and/or providing in-home breastfeeding support.

## Data Availability

The dataset or transcripts are available from the corresponding author on reasonable request.
